# Time-dependent effects of endogenous hyperglucagonemia on glucose homeostasis and hepatic glucagon action

**DOI:** 10.1172/jci.insight.162255

**Published:** 2023-06-08

**Authors:** Camila Lubaczeuski, Nadejda Bozadjieva-Kramer, Ruy A. Louzada, George K. Gittes, Gil Leibowitz, Ernesto Bernal-Mizrachi

**Affiliations:** 1 Department of Medicine, Division Endocrinology, Metabolism and Diabetes, Miller School of Medicine, University of Miami, Miami, Florida, USA.; 2 Veterans Affairs Ann Arbor Healthcare System, Research Service, Ann Arbor, Michigan, USA.; 3 Department of Surgery, University of Michigan, Ann Arbor, Michigan, USA.; 4 Childrens Hospital, University of Pittsburgh School of Medicine, Pittsburgh, Pennsylvania, USA.; 5 Diabetes Unit and Endocrine Service, Hadassah-Hebrew University Medical Center, Jerusalem, Israel.; 6 Veterans Affairs Medical Center, Miami, Florida, USA.

**Keywords:** Endocrinology, Metabolism, Glucose metabolism, Islet cells

## Abstract

Elevation of glucagon levels and increase in α cell proliferation is associated with states of hyperglycemia in diabetes. A better understanding of the molecular mechanisms governing glucagon secretion could have major implications for understanding abnormal responses to hypoglycemia in patients with diabetes and provide novel avenues for diabetes management. Using mice with inducible induction of Rheb1 in α cells (αRheb^Tg^ mice), we showed that short-term activation of mTORC1 signaling is sufficient to induce hyperglucagonemia through increased glucagon secretion. Hyperglucagonemia in αRheb^Tg^ mice was also associated with an increase in α cell size and mass expansion. This model allowed us to identify the effects of chronic and short-term hyperglucagonemia on glucose homeostasis by regulating glucagon signaling in the liver. Short-term hyperglucagonemia impaired glucose tolerance, which was reversible over time. Liver glucagon resistance in αRheb^Tg^ mice was associated with reduced expression of the glucagon receptor and genes involved in gluconeogenesis, amino acid metabolism, and urea production. However, only genes regulating gluconeogenesis returned to baseline upon improvement of glycemia. Overall, these studies demonstrate that hyperglucagonemia exerts a biphasic response on glucose metabolism: Short-term hyperglucagonemia lead to glucose intolerance, whereas chronic exposure to glucagon reduced hepatic glucagon action and improved glucose tolerance

## Introduction

Type 1 and type 2 diabetes are characterized by uncontrolled hyperglycemia associated with the progressive failure of pancreatic islet β cells and, ultimately, a reduction in insulin levels. Clinical evidence also demonstrates that elevation of glucagon levels in type 2 diabetes may contribute to the pathogenesis of hyperglycemia by enhancing hepatic glucose output (1–6). In addition to the contribution to hyperglycemia, the failure of α cells to secrete glucagon in response to hypoglycemia is a major limiting factor for optimal glucose control in patients with type 1 diabetes (7) or advanced type 2 diabetes (8, 9). Thus, a better understanding of the molecular mechanisms governing glucagon secretion and action and its effect on glycemia has important implications for the pathophysiology of diabetes.

Stimulation of glucagon secretion in hypoglycemia induces hepatic glucose production via cellular mechanisms, including suppression of glycogenesis and glycolysis and stimulation of glycogenolysis and gluconeogenesis (10). In addition to low glucose, amino acids have been shown to induce glucagon secretion (11). Postprandial elevation of circulating amino acids has been observed after a high-protein meal, and this is exacerbated by chronic protein consumption in rodents and humans (12–14). The close link between amino acids and the α cell is highlighted by the liver–α cell axis. This axis was identified by the major increase in α cell hyperplasia and hyperglucagonemia in models of reduced glucagon action in hepatocytes genetically or pharmacologically by treatment with glucagon receptor antagonists (GRAs), which was subsequently attributed to the dramatic rise in amino acids (15–18). Importantly, hyperglucagonemia induced by hyperaminoacidemia after treatment with GRA is mediated at least in part by mTORC1 (17, 18, 19). Induction of mTORC1 by constitutive genetic deletion of TSC2 in α cells (αTSC2^KO^) recapitulated the effects of chronic hyperaminoacidemia with increases in α cell mass and development of chronic hyperglucagonemia indicating that mTORC1 mediates amino acids signals in α cells (20). We previously showed that chronic hyperglucagonemia in αTSC2^KO^ animals resulted in development of hepatic glucagon resistance with subsequent improvement of glucose tolerance (20). In contrast, inhibition of mTORC1 signaling in α cells decreases glucagon content and glucagon secretion in response to different secretagogues (19). While these studies highlight the important role of mTORC1 in α cell function and the potential link between chronic hyperglucagonemia and liver glucagon resistance, it is unclear if short-term (3–10 days) stimulation of mTORC1 can increase glucagon secretion irrespective of α cell mass and whether hyperglucagonemia-induced glucagon resistance is reversible.

The current studies were aimed at assessing the effects of short- versus long-term induction of mTORC1 signaling on glucagon secretion and action, α cell mass, and glucose tolerance and the reversibility of these alterations. Using mice with inducible and reversible activation of mTORC1 in α cells, we show that chronic hyperglucagonemia improved glucose homeostasis through effects on hepatic glucagon receptor (GCGR) expression and hepatic glucagon signaling. These studies demonstrate that changes in glucagon levels and the duration of hyperglucagonemia can impact glucose homeostasis by reducing glucagon action in the liver.

## Results

### Animal model of inducible hyperglucagonemia by overexpression of Rheb in α cells.

**Figure 1:**
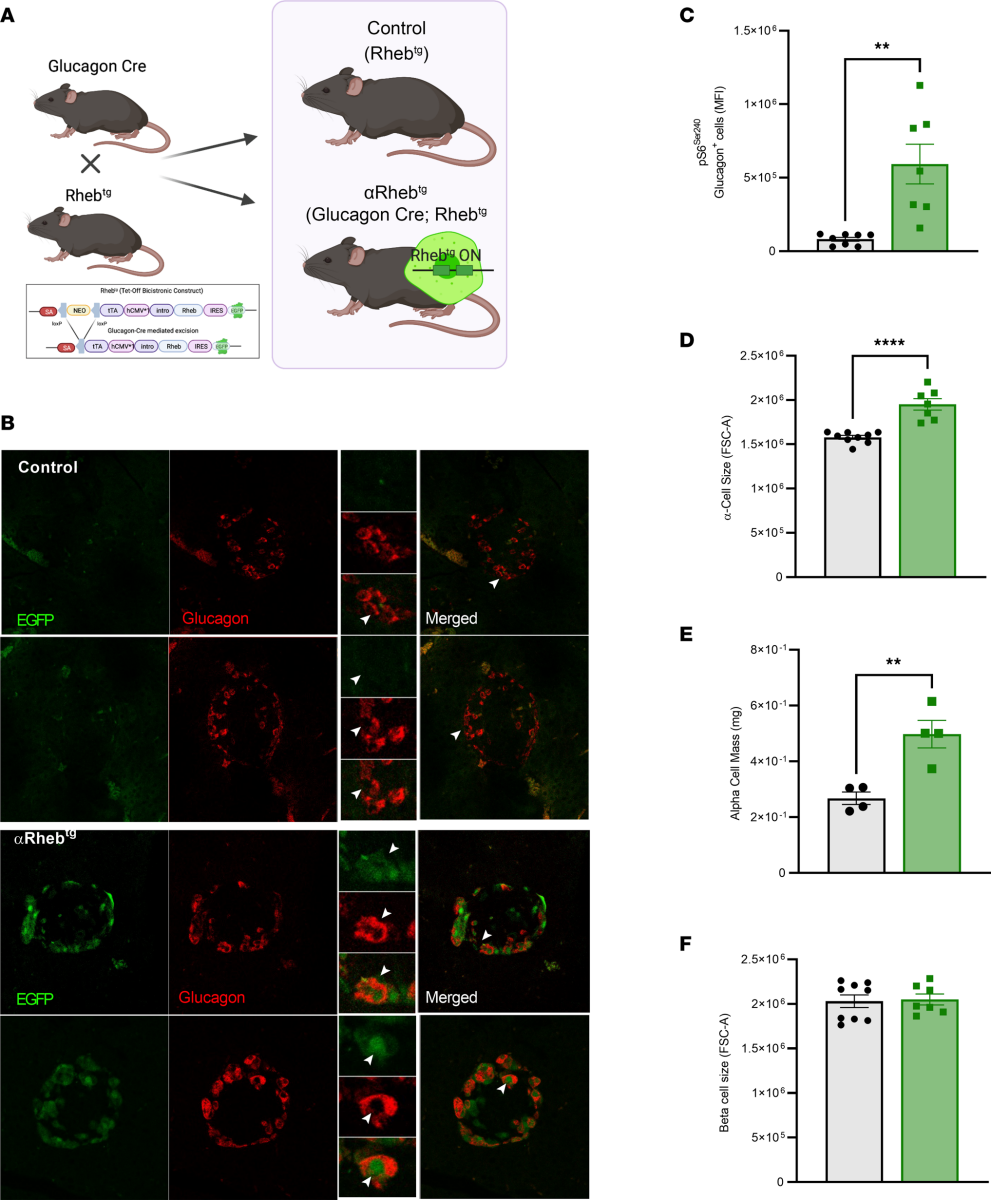
Animal model of inducible hyperglucagonemia by overexpression of Rheb in α cells. (**A**) Conditional targeting strategy for the generation of Rheb^Tg^ mice. (**B**) Representative images from pancreas sections stained for glucagon and endogenous GFP (EGFP) in 3-month-old αRheb^Tg^ and controls (Rheb^Tg^). Arrowheads denote α cells. Original magnification, ×20. (**C**) Assessment of pS6^Ser240^ by MFI measured by flow cytometry in dispersed α cells (*n* = 7 controls and *n* = 6 αRheb^Tg^). (**D**) α cell size measured in dispersed islets from control (*n* = 9) and αRheb^Tg^ (*n* = 7) mice at 3 months of age. Cell size was analyzed by flow cytometry and quantified by forward scatter area (FSC-A). (**E**) α Cell mass in control (*n* = 4) and αRheb^Tg^ (*n* = 4) mice at 3 months of age. (**F**) β Cell size analyzed by flow cytometry using dispersed islets and quantified by FSC-A of control (*n* = 9) and αRheb^Tg^ (*n* = 7) mice at 3 months of age. Data are shown as the mean ± SEM. **P < 0.01, ***P < 0.001, ****P < 0.001 (Student’s 2-tailed *t* test).

To induce mTORC1 signaling in α cells, we used a transgenic model with inducible Rheb overexpression. This transgenic model overexpresses wild-type Rheb using a single-cassette inducible system (Tet-off/expression suppressed by doxycycline [Dox]) (Figure 1A). Overexpression of a wild-type Rheb is sufficient to activate mTORC1 signaling (21). In this system, once the tetracycline trans activator (tTA) gene is activated by *Cre*-mediated recombination, tTA binds to Tet binding sites and induces Rheb and EGFP expression under a generalized promoter. Therefore, Rheb expression in these mice will be repressed in the presence of Dox but induced upon withdrawal of Dox. Rheb overexpression in pancreatic α cells was achieved by crossing Glucagon-Cre and Rheb homozygous transgenic (αRheb^Tg^) mice (Figure 1A). Overexpression of transgenic Rheb (Rheb^Tg^) primarily in α cells is shown by costaining for glucagon and EGFP in pancreatic sections (Figure 1B). The effect of Rheb overexpression on mTORC1 activity was validated by an increase in pS6 (Ser240) staining in sorted α cells from αRheb^Tg^ mice, a surrogate marker for activation of the Rheb/mTORC1 axis (Figure 1C). αRheb^Tg^ mice also exhibited a significant increase in α cell mass (Figure 1E), and this was accompanied by an increase in α cell size analyzed by flow cytometry and quantified by forward scatter area (FSC-A) in dispersed islets (Figure 1D). The increase in α cell mass is likely to occur postnatally, as neonatal α cell mass is conserved in models of gain or loss of mTORC1 (19, 20). The increased α cell size observed in αRheb^Tg^ mice was further supported by increased mTORC1 activity, as this kinase positively regulates cell size by activation of S6K (22, 23). Importantly, the activation of mTORC1 in α cells did not lead to changes in β cell size in αRheb^Tg^ mice compared with control mice (Figure 1F).

### Time-dependent changes in glucose homeostasis after chronic hyperglucagonemia in αRheb^Tg^ mice.

**Figure 2:**
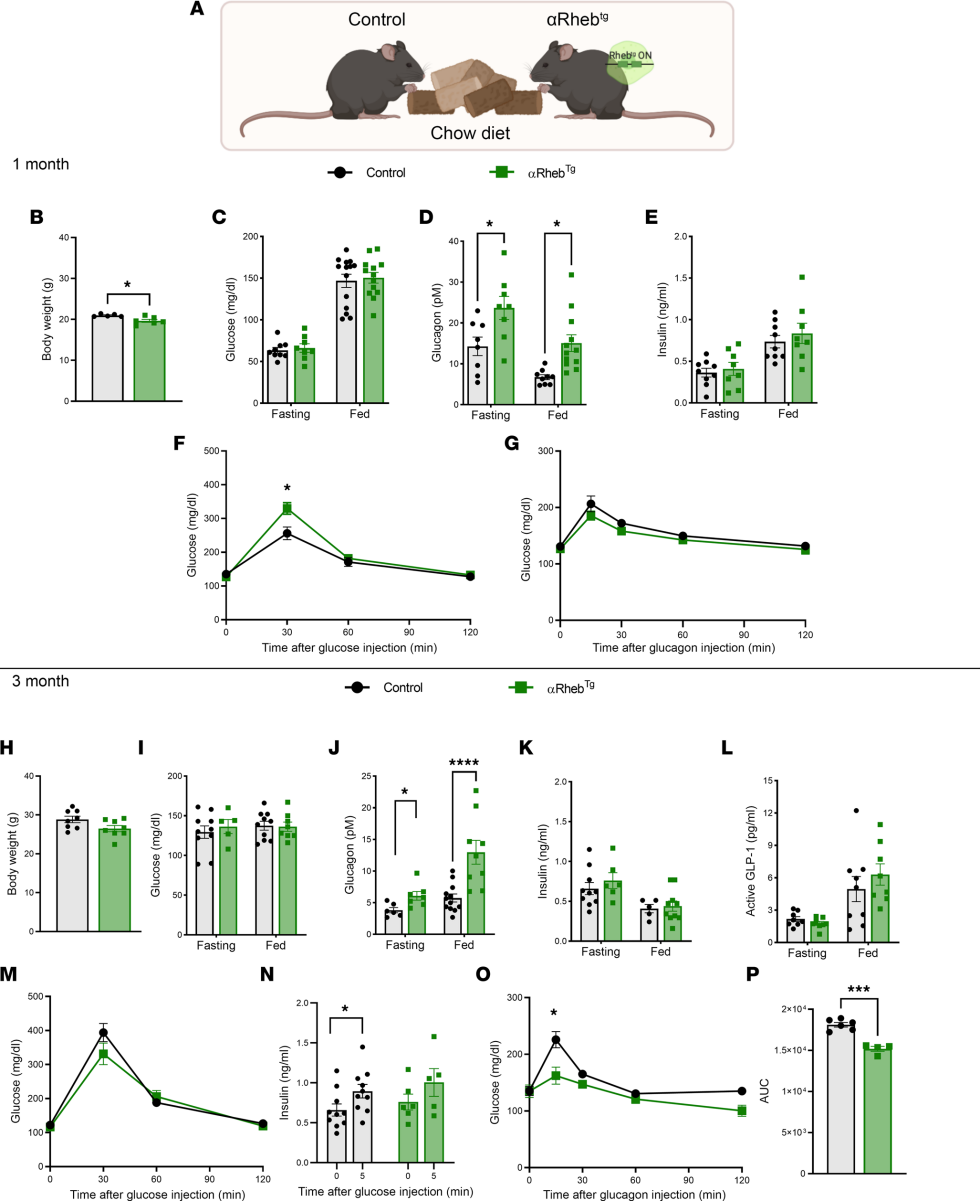
Time-dependent changes in glucose homeostasis after chronic hyperglucagonemia in αRheb^Tg^ mice. (**A**) Control or αRheb^Tg^ mice were exposed to chow diet during pregnancy and postnatally for 3 months**.** (**B**) Body weight of 1-month-old control (*n* = 5) and αRheb^Tg^ (*n* = 6) mice. (**C**) Blood glucose (*n* = 8–9), (**D**) glucagon (*n* = 8–11), and (**E**) insulin were measured after 16 hours of fasting or feeding in 1-month-old control and αRheb^Tg^ (*n* = 8–9) mice**.** (**F**) Glucose tolerance test (2 g/kg.bw) in 1-month-old control (*n* = 11) or αRheb^Tg^ (*n* = 14) mice and (**G**) glucagon tolerance test (100 μg/kg) in 1-month old control (*n* = 9) or αRheb^Tg^ (*n* = 8) mice. (**H**) Body weight of 3-month-old control (*n* = 8) and αRheb^Tg^ (*n* = 8) mice. (**I**) Blood glucose (*n* = 5–10), (**J**) glucagon (*n* = 6–12), (**K**) insulin (*n* = 5–10), and (**L**) active GLP-1 (*n* = 8–9) were measured after 16 hours of fasting or feeding in 3-month-old control and αRheb^Tg^ mice. (**M**) Glucose tolerance test (2 g/kg.bw) in 3-month-old control (*n* = 5) or αRheb^Tg^ (*n* = 8) mice and (**N**) glucose-stimulated insulin secretion (3 g/kg.bw) in 3-month-old control (*n* = 10) and αRheb^Tg^ (*n* = 5–6) mice. (**O**) Glucose response to intraperitoneal glucagon (100 g/kg), and (**P**) area under the curve (AUC) in 3-month-old control (*n* = 6) or αRheb^Tg^ (*n* = 4) mice. For **B**–**E**, **H**–**L**, **N**, and **P,** data are shown as mean ± SEM. *P < 0.05, ***P < 0.001, ****P < 0.0001 (Student’s 2-tailed *t* test). For **F**, **G**, **M**, and **O,** data are shown as mean ± SEM. *P < 0.05 (2-way ANOVA with Šidák’s post test).

We first assessed the effects of mTORC1 activation in αRheb^Tg^ mice on regular chow diet during pregnancy and the first month of life (Figure 2A). At 1 month of age, αRheb^Tg^ mice had lower weight compared with controls, but this difference was not observed at 3 months of age (Figure 2, B and H). Despite being normoglycemic in the fasting and fed state, αRheb^Tg^ mice had increased glucagon and normal insulin levels (Figure 2, C–E). However, when challenged with intraperitoneal glucose, 1-month-old αRheb^Tg^ mice exhibited higher glucose levels at 30 minutes after glucose injection and comparable responses after glucagon administration (Figure 2, F and G). At 3 months of age, αRheb^Tg^ and control mice had comparable body weight (Figure 2H). While higher glucagon levels were observed in 3-month-old αRheb^Tg^ mice, these mice displayed similar fed and fasting glucose, insulin, glucagon, and glucose tolerance when compared with controls (Figure 2, I–K and M). Glucose-stimulated insulin secretion in vivo and ex vivo was comparable between the groups (Figure 2N and Supplemental Figure 2D; supplemental material available online with this article; https://doi.org/10.1172/jci.insight.162255DS1). This suggests that the glucose intolerance observed in 1-month-old αRheb^Tg^ mice was lost by 3 months. In contrast to that in 1-month-old αRheb^Tg^ mice, glucose excursion during the glucagon tolerance test showed diminished responses in αRheb^Tg^ mice to exogenous glucagon at 100 μg/kg (Figure 2, O and P) and 20 μg/kg (Supplemental Figure 2, A and B). No changes in fasting and fed GLP-1 levels were observed among the groups (Figure 2L). The lack of glucose intolerance after chronic elevation of glucagon in αRheb^Tg^ mice and the reduced responses to glucagon administration recapitulate the glucagon resistance phenotype observed in mice with constitutive hyperglucagonemia by constitutive deletion of TSC2 in α cells (20). Further support for a decrease in glucagon action in the αRheb^Tg^ liver with chronic elevation of glucagon is supported by decreased hepatic glucagon signaling measured by phosphor-CREB (Supplemental Figure 2C). No changes in liver responses to insulin were seen with phospho-AKT signaling (Supplemental Figure 3E).

### Postnatal induction of hyperglucagonemia results in transient fasting hyperglycemia and glucose intolerance.

**Figure 3:**
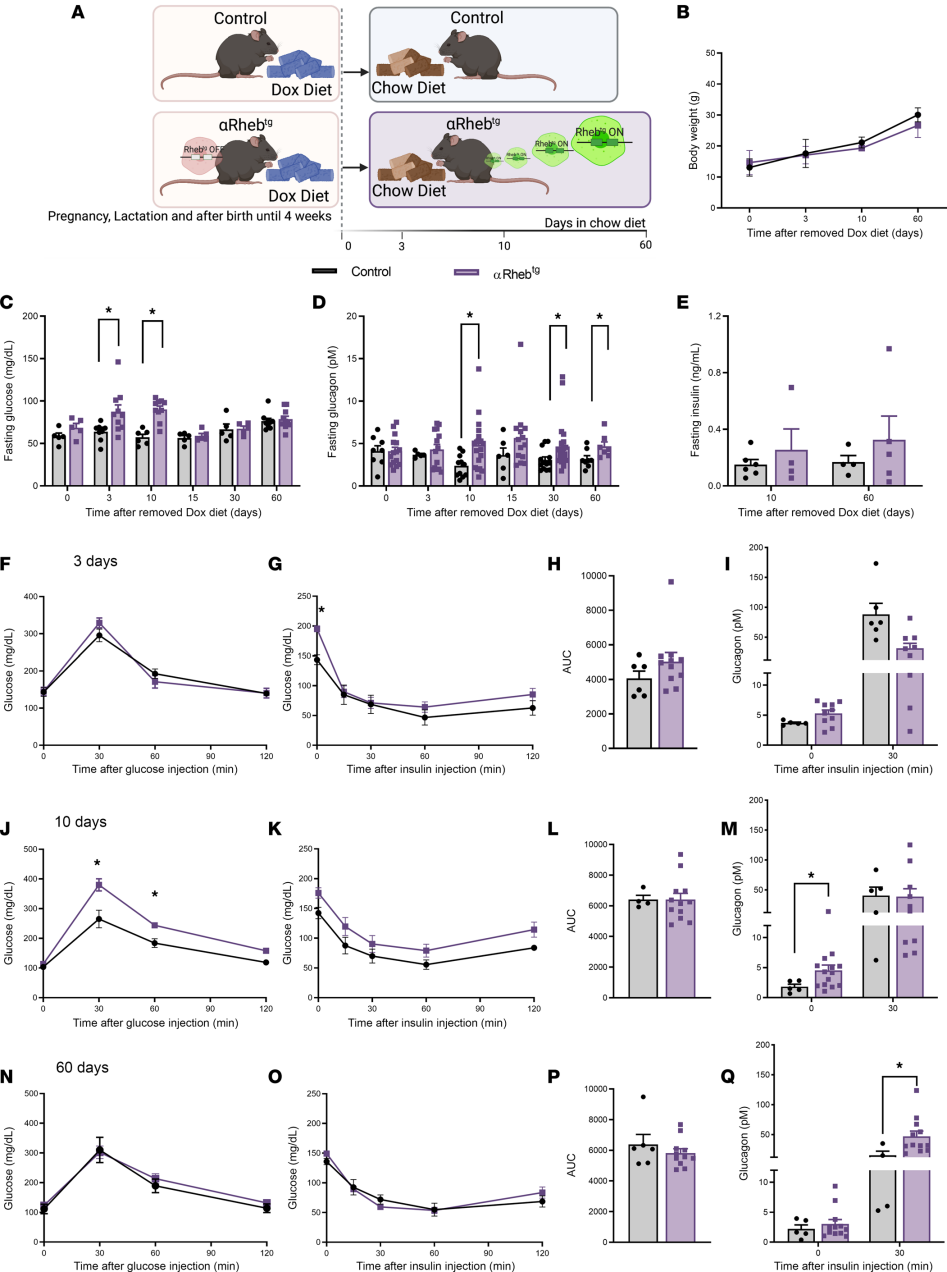
Postnatal induction of hyperglucagonemia results in transient fasting hyperglycemia and glucose intolerance. (**A**) Control and Rheb^Tg^ mice were exposed to Dox diet during pregnancy, lactation, and weaning. After 1 month of age, control and αRheb^Tg^ mice were placed on regular chow to induce Rheb overexpression. (**B**) Body weight before removing Dox (day 0) and after 3, 10, and 60 days of switching to control chow (*n* = 11 controls and *n* = 14 αRheb^Tg^). (**C**) Fasting (12 hours) glucose (*n* = 5–10 controls and *n* = 5–10 αRheb^Tg^) and (**D**) fasting (12 hours) glucagon levels before removing Dox and switching to control chow (day 0) (*n* = 6–17 controls and *n* = 7–22 αRheb^Tg^). (**E**) Fasting (12 hours) insulin levels after removing Dox (day 10 and day 60) in control (*n* = 3–4) and αRheb^Tg^ (*n* = 4–6) mice. (**F**) i.p. glucose tolerance test (2 g/kg.bw) (*n* = 9 controls and *n* = 8 αRheb^Tg^) after 6-hour fast. (**G**) Blood glucose response during ITT (0.75 units/kg.bw) and (**H**) AUC calculated for the ITT normalized by the baseline (*n* = 6–8 controls and *n* = 14–11 αRheb^Tg^). (**I**) Glucagon response before (4-hour fast) and after 30 minutes of insulin injection (0.75 units/kg.bw) performed 3 days after removing Dox diet (*n* = 5–6 controls and n= 9 αRheb^Tg^). (**J**) Glucose tolerance test (2 g/kg.bw) (*n* = 7–9 controls and *n* = 13 αRheb^Tg^) after 6-hour fast. (**K**) Blood glucose response to ITT (0.75 units/kg.bw) and (**L**) AUC calculated for the ITT normalized by the baseline (*n* = 5 controls and *n* = 13 αRheb^Tg^). (**M**) Glucagon response before (4-hour fast) and after 30 minutes of insulin injection (0.75 units/kg.bw) performed 10 days after in control chow diet (*n* = 4–5 controls and *n* = 9–14 αRheb^Tg^). (**N**) Glucose tolerance test (2 g/kg.bw) performed 60 days after removing Dox diet (*n* = 4 controls and *n* = 7 αRheb^Tg^) after 6-hour fast. (**O**) Blood glucose response to ITT (0.75 units/kg.bw) and (**P**) AUC calculated for the ITT normalized by the baseline (*n* = 6–9 controls and *n* = 11 αRheb^Tg^). (**Q**) Glucagon response before (4-hour fast) and after 30 minutes of insulin injection (0.75 units/kg.bw) performed 60 days after removing Dox diet (*n* = 4–5 controls and *n* = 11–12 αRheb^Tg^). Data for **B**, **F**, **G**, **J**, **K**, **N**, and **O** are shown as the mean ± SEM. *P < 0.05 (2-way ANOVA with Šidák’s post test), and for **C**, **D**, **E**, **H**, **I,** **L**, **M**, **P**, and **Q,** data are shown as the mean ± SEM. *P < 0.05 (Student’s 2-tailed *t* test).

To determine the effect of overexpressing Rheb in α cells after the maturation/development phase, Dox diet was administered to control and αRheb^Tg^ mice during pregnancy and 30 days after birth followed by administration of control chow (Figure 3A). Examination of α cells at 10 days after removing Dox shows that postnatal activation of mTORC1 led to increased pS6 levels (Supplemental Figure 3C) and increased α cell number (Supplemental Figure 3A), without changes in glucagon expression and α cell size (Supplemental Figure 3, B and D). Given the known expression of *Cre* recombinase in the central nervous system using this Glucagon-Cre model (19, 20), we assessed weight changes after mTORC1 activation. No changes in weight were observed in αRheb^Tg^ mice (Figure 3B). Glucose and glucagon levels after 12-hour fast were similar between the groups at P30 before switching to control chow (day 0, Figure 3, C–E). Short-term mTORC1 activation in αRheb^Tg^ mice increased fasting glycemia as early as day 3 after removing Dox from the diet (Figure 3C). Glucose levels in 12-hour-fasted αRheb^Tg^ mice returned to normal at 15 days after Dox removal (Figure 3C). The changes in glucose after 12 hours of fasting were accompanied by higher glucagon levels at 10, 30, and 60 days of Dox removal, with no change in 12-hour fasting insulin (Figure 3, C–E). Overexpression of Rheb in α cells for 3 days did not affect the glucose tolerance in Rheb^Tg^ mice (Figure 3F). In contrast to the normal glucose after 6 hours of fasting (time 0 IPGTT, Figure 3F), αRheb^Tg^ mice exhibited hyperglycemia after 4-hour fast (time 0, Figure 3G). Insulin sensitivity was conserved after Rheb overexpression for 3 days in αRheb^Tg^ mice (Figure 3, G and H). Additionally, no changes were observed in 4-hour fasting glucagon or glucagon secretion after insulin-induced hypoglycemia (Figure 3I). After 10 days of Rheb overexpression, αRheb^Tg^ mice showed impaired glucose tolerance and comparable insulin tolerance when compared with controls (Figure 3, J–L). Although αRheb^Tg^ mice showed higher glucagon levels after 4-hour fast, the glucagon response to hypoglycemia was comparable to that of controls (Figure 3M). Sixty days of Rheb overexpression and hyperglucagonemia resulted in normalization of glucose tolerance in αRheb^Tg^ mice, and this was accompanied by conserved insulin sensitivity (Figure 3, N–P). When fasted for 4 hours (Figure 3Q), glucagon levels were comparable between αRheb^Tg^ mice and controls, in contrast to the increased glucagon levels in αRheb^Tg^ mice after 12-hour fast (Figure 3D). However, glucagon secretion by insulin-induced hypoglycemia was enhanced in αRheb^Tg^ mice, suggesting the presence of impaired hepatic glucagon action in αRheb^Tg^ mice (Figure 3Q).

### Hyperglucagonemia in αRheb^Tg^ mice is reversible after turning off Rheb expression with Dox treatment.

**Figure 4:**
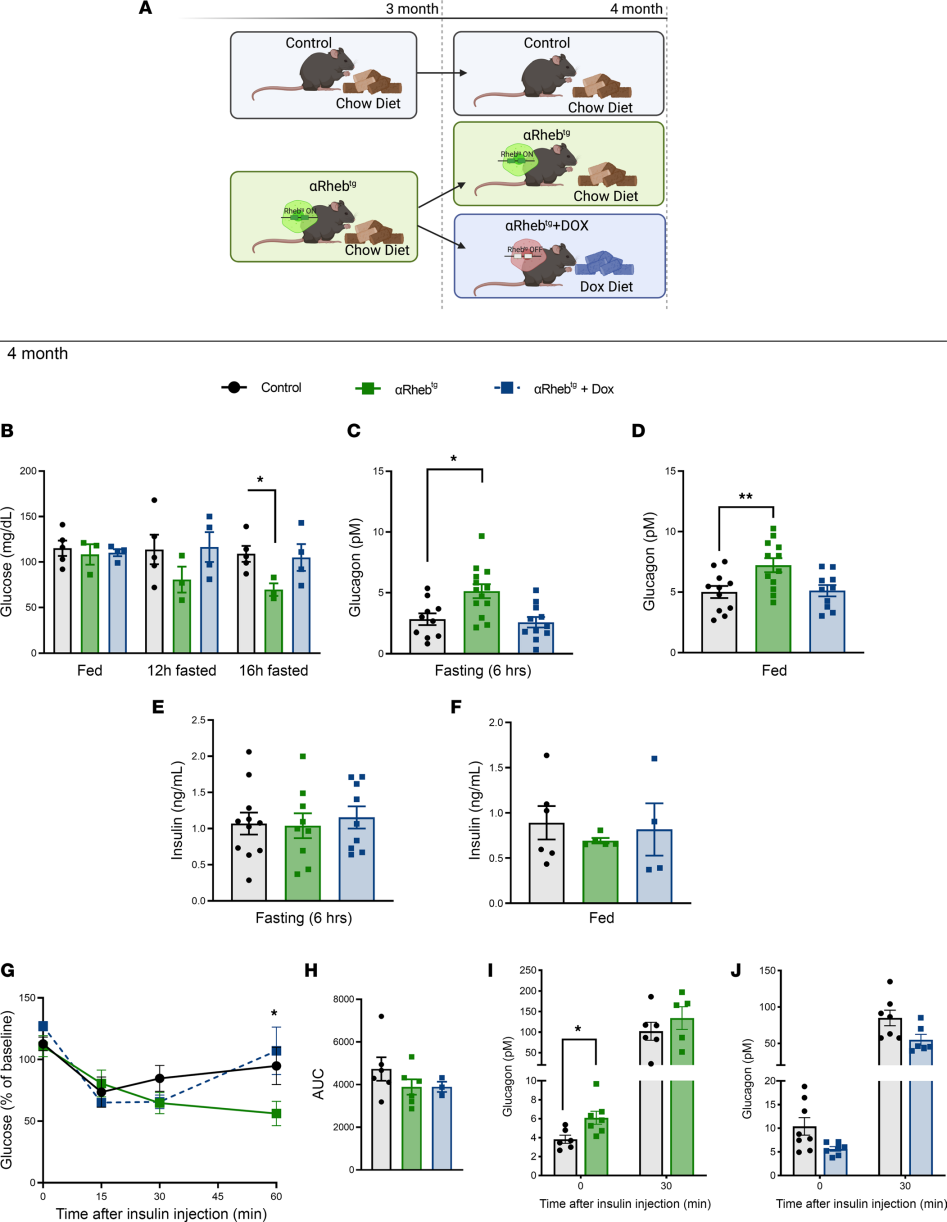
Hyperglucagonemia in αRheb^Tg^ mice is reversible after turning off Rheb expression with Dox treatment. (**A**) Control or αRheb^Tg^ mice were exposed to chow diet after weaning until 3 months of age when half of the αRheb^Tg^ mice were switched to Dox diet (23%, 200 mg Dox/kg) for 1 month and the other half remained on control chow. (**B**) Changes in glucose levels in fed, 12-hour fasted, or 16-hour fasted mice (*n* = 3–5). (**C**) Fasting glucagon levels (6 hours) (*n* = 10–13) and (**D**) fed glucagon levels (*n* = 10–12). (**E**) Fasting insulin levels (6 hours) (*n* = 9–11) and (**F**) fed insulin levels (*n* = 4–6). (**G**) Blood glucose response to ITT (0.75 units/kg.bw) in 4-month-old controls (*n* = 6), αRheb^Tg^ mice (*n* = 6), and 4-month-old αRheb^Tg^+Dox mice (*n* = 3). (**H**) AUC calculated for the ITT in 4-month-old controls (*n* = 6), αRheb^Tg^ mice (*n* = 6), and 4-month-old αRheb^Tg^+Dox mice (*n* = 3). (**I**) Glucagon response to hypoglycemia induced by insulin (0.75 unit/kg.bw) in 3-month-old controls (*n* = 6–8), αRheb^Tg^ mice (*n* = 5–7), and (**J**) 4-month-old αRheb^Tg^+Dox mice (*n* = 6–7). Data are shown as the mean ± SEM. (**B**–**D** and **G**) *P < 0.05 (2-way ANOVA with Tukey’s post test). (**I**) *P < 0.05 (Student’s 2-tailed *t* test).

Next, we assessed if hyperglucagonemia and associated changes in glucose metabolism observed in αRheb^Tg^ mice were reversible. For these studies, we overexpressed Rheb during development and postnatally by feeding regular chow to αRheb^Tg^ and control mice during pregnancy and 3 months after birth (Figure 4A). At 3 months, Rheb overexpression was suppressed in half of αRheb^Tg^ mice by switching to Dox diet for 4 weeks (Figure 4A). The other half of αRheb^Tg^ mice and controls were kept in control chow for 4 weeks to complete 4 months of Rheb overexpression (Figure 4A). Examination of fasting glucose at 4 months showed that αRheb^Tg^ mice on regular chow had lower glucose after 12-hour fast, and these glucose levels became significant after a 16-hour fast (Figure 4B). In contrast, αRheb^Tg^ mice in which Rheb expression was turned off by Dox administration (αRheb^Tg^+Dox) exhibited fasting glucose comparable to that of controls (Figure 4B) with no changes in body weight (Supplemental Figure 4A). Glucagon levels were higher in 6-hour fasting and fed αRheb^Tg^ mice on control chow (Figure 4, C and D). The decrease in glucose levels in αRheb^Tg^ mice on control chow was not explained by changes in insulin levels in the fed or fasting state (Figure 4, E and F). Blood glucose levels after exogenous insulin were similar in controls and αRheb^Tg^+Dox mice 60 minutes after insulin administration (Figure 4, G and H). In contrast, hyperglucagonemic αRheb^Tg^ displayed lower glucose levels 60 minutes after insulin injection (Figure 4, G and H). These results suggest that chronic hyperglucagonemia induces lower fasting glucose levels but not the glucagon responses to insulin-induced hypoglycemia (Figure 4, I and J). The lower glucose at 60 minutes after insulin injection in hyperglucagonemic αRheb^Tg^ mice is reminiscent of results observed in αTSC^KO^ mice and suggests a decrease in glucagon action in the liver (20). Overall, these data showed that hyperglucagonemia and associated changes in glucose metabolism observed in αRheb^Tg^ mice were reversible upon discontinuation of Rheb activation in α cells.

### Downregulation of GCGR expression and gluconeogenic genes by chronic hyperglucagonemia is reversed after normalization of glucagon levels.

**Figure 5:**
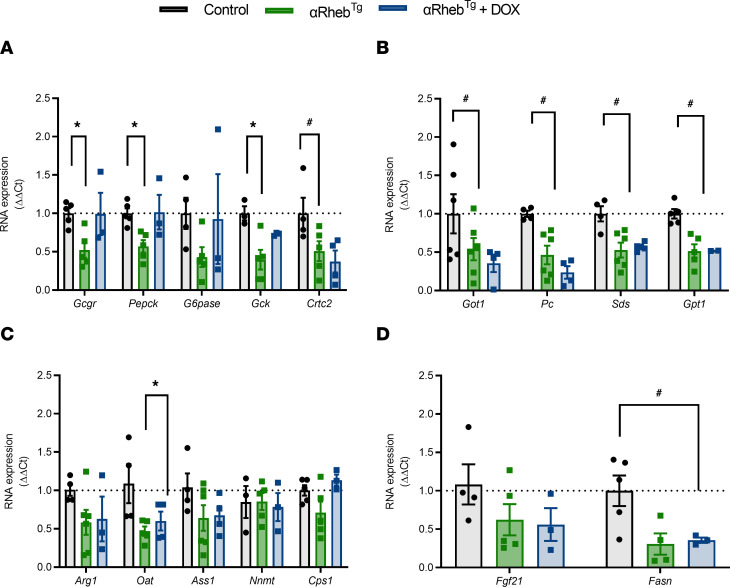
Downregulation of GCGR expression and gluconeogenic genes by chronic hyperglucagonemia is reversed after normalization of glucagon levels. RNA expression of hepatic key enzymes involved in (**A**) gluconeogenesis, (**B**) amino acid metabolism, and (**C**) urea metabolism in 6-hour-fasted liver from control (*n* = 3–5), αRheb^Tg^ (*n* = 5), and αRheb^Tg^+Dox (*n* = 3–4) mice. (**D**) *Fgf21* and fatty acid synthase (*Fasn*) in 6-hour-fasted liver from control (*n* = 4–5), αRheb^Tg^ (*n* = 5–4), and αRheb^Tg^+Dox mice. Data for **A**–**D** are shown as the mean ± SEM. * means significant differences between control and αRheb^Tg^ and # means differences between controls versus αRheb^Tg^ and αRheb^Tg^+Dox mice (*n* = 3). *P < 0.05 (1-way ANOVA with Dunnett post test). **P < 0.01 (Student’s 2-tailed *t* test).

To determine if hyperglucagonemia and associated changes in glucose metabolism in αRheb^Tg^ mice were caused by downregulation of GCGR, we assessed the hepatic expression of GCGR and genes involved in glucagon signaling and gluconeogenesis in the liver using the same experimental design described in Figure 4A. These studies demonstrated that hepatic mRNA expression of *Gcgr*, phosphoenolpyruvate carboxykinase (*Pepck*), and glucokinase (*Gck*) was decreased in αRheb^Tg^ mice with chronic hyperglucagonemia, which may suggest that glycolysis is also impaired (Figure 5A). Expression of these genes was normalized once glucagon levels returned to normal after Dox diet administration in αRheb^Tg^ mice (αRheb^Tg^+Dox) (Figure 5A). A similar pattern was observed for glucose 6-phosphatase (*G6pase*) but did not achieve statistical differences (Figure 5A). CREB regulated transcription coactivator 2 (*Crtc2*) and *Fasn* mRNA expression were decreased in αRheb^Tg^ mice but were not recovered after Dox-diet administration (Figure 5, A–D). No changes were observed in liver mRNA levels of *Fgf21* (Figure 5D). Another important action of glucagon in the liver is the increase in amino acid uptake, metabolism, and urea production (24). Assessment of hepatic genes involved in amino acid metabolism showed downregulation of *Got1*, *Pc*, *Sds*, and *Gpt1* mRNA in fasted Rheb^Tg^ ^mice^, and those genes were not recovered after Dox-diet administration in αRheb^Tg^+Dox (Figure 5B). Examination of genes involved in urea metabolism showed decreased mRNA expression in *Oat* in fasted liver from αRheb^Tg^ mice, and this was no different in αRheb^Tg^+Dox mice. No changes in mRNA expression were observed in *Arg1*, *Ass1*, *Cps1*, and *Nnmt* (Figure 5C). Urea production and L-amino acid levels were comparable in αRheb^Tg^ and αRheb^Tg^+Dox mice after fasting, indicating that amino acid metabolism was not changed in these mice (Supplemental Figure 2, B and C).

## Discussion

Increased glucagon levels have been linked to the pathogenesis of hyperglycemia in type 2 diabetes. There is less understanding about the effects of chronic endogenous hyperglucagonemia on glucose homeostasis (25). Using a model of inducible hyperglucagonemia caused by mTORC1 activation in α cells, our studies uncovered a potentially novel biphasic response characterized by an early phase of glucose intolerance followed by a phase of reduction in liver glucagon action and restoration of glucose tolerance. We also discovered that hyperglucagonemia and associated reduction in glucagon activity in the liver are reversible after normalization of glucagon levels following cessation of mTORC1 activation in α cells. We believe these findings are critical to interpreting the temporal changes in glucose homeostasis after increases in glucagon in diabetes. At the same time, these findings are important considering the current clinical trials exploring the use of dual insulin/glucagon pumps for the treatment of diabetes.

We demonstrated that the αRheb^Tg^ mouse is a suitable model to study reversible hyperglucagonemia in physiology and disease states. While this Glucagon-Cre model induces recombination in L cells and in the brain stem and hypothalamic regions (dorsomedial hypothalamic nucleus) (26, 27) the levels of mTORC1 activation achieved in αRheb^Tg^ mice were insufficient to induce changes in weight and GLP-1 levels (Figures 2, B, H, and L). Using this model, we showed that short-term mTORC1 activation (3–10 days) in α cells rapidly stimulates glucagon secretion (Figure 3D), followed by increased α cell number over time (Supplemental Figure 3, A and C) (28). The short-term hyperglucagonemia led to fasting hyperglycemia along with glucose intolerance during the first 10 days following mTORC1 activation (Figure 3, C, D, and J). At this stage, hyperglucagonemia increased glucose levels by gluconeogenesis and increased in glucose output by the liver. Interestingly, the short-term induction by 3 days caused increases in fasting glucose without concomitant elevation of glucagon levels in circulation (Figure 3, C and D). The mechanisms for this observation are not completely clear, but we propose that mild increases in glucagon during the first 3 days are sufficient to induce hyperglycemia due to increased glucose output, but glucagon levels during this early phase are below the saturation threshold of hepatic clearance mechanism (receptor binding) and most glucagon is cleared during the first pass through the liver (29). Further increases in glucagon levels after 3 days overcome the hepatic clearance mechanisms, resulting in augmented glucagon levels in the circulation (Figure 3D). Further studies could be designed to assess the adaptation of hepatic glucagon clearance to chronic hyperglucagonemia.

These studies showed that, in the long-term phase (60 days), chronic hyperglucagonemia leads to normalization of glucose tolerance in αRheb^Tg^ mice, at least in part, by downregulation of hepatic *Gcgr* expression associated with decreased phosphor-CREB levels and transcription of hepatic genes involved in glucagon signaling (Figure 3N, Figure 5A, and Supplemental Figure 2C). Importantly, these changes were associated with conserved insulin sensitivity as demonstrated by insulin tolerance test (ITT) and normal hepatic Akt phosphorylation responses to insulin stimulation (Supplemental Figure 2E). The lack of changes in insulin sensitivity induced by progressive and long-term high glucagon levels is in contrast with previous work showing that acute *Gcgr* agonism (few hours) stimulates insulin signaling in the liver (30). These findings suggest that acute glucagon effects on insulin sensitivity could be lost in conditions of prolonged hyperglucagonemia. In addition to a decrease in glucagon action in the liver, it is possible that insulinotropic effects of glucagon on the β cells could contribute to normalization of glucose tolerance (31). However, the normal in vivo and ex vivo insulin secretory responses to glucose in αRheb^Tg^ mice (Figure 2N, Figure 3E, Figure 4, E and F, and Supplemental Figure 2D) indicate that the glucagon levels achieved in this model are insufficient to induce insulin secretion. In summary, these results are important because together they suggest that the effects of glucagon on glucose homeostasis depend on the levels and duration of hyperglucagonemia.

Using the αRheb^Tg^ model, we showed that reduction in hepatic glucagon activity is reversible upon normalization of glucagon levels (Figure 5, A and B). Hepatic resistance to glucagon action has been reported since 1970 (32–35). This molecular phenomenon is characterized by impaired physiological effects of glucagon, including glucose responses, cAMP levels, glycogen breakdown, glucose production, and amino acid and lipid metabolism. Other studies have included glucagon binding to the receptor and expression of liver GCGR at the mRNA and protein levels. The decrease in hyperglycemic responses to glucagon in vivo included reduced *Gcgr*, gluconeogenic genes, and genes involved in amino acid metabolism in the liver as well as amelioration of hepatic CREB phosphorylation and hyperglycemic responses after stimulation with glucagon, which are consistent with the development of partial glucagon resistance in αRheb^Tg^ mice, as described in some of the published literature (32–35). Given that the expression of liver GCGR is partial, it is possible that this model does not recapitulate all the effects observed in mice with deletion of GCGR in the liver (16, 36). The current studies showed that chronic hyperglucagonemia in αRheb^Tg^ mice downregulates *Gcgr* expression in the liver to a lesser extent than in αTSC2^KO^ mice (for example, 76% reduction in the αTSC2^KO^ [ref. 20] versus 48% in αRheb^Tg^ mice, Figure 5A). This is likely explained by the significantly higher glucagon levels in αTSC2^KO^ compared with αRheb^Tg^ mice (αTSC2^KO^ > 25 pM and αRheb^Tg^ = 7–10 pM fasting glucagon levels) (20). The magnitude of hyperglucagonemia and reduction of *Gcgr* expression obtained in the inducible adult αRheb^Tg^ mice is more aligned to glucagon levels in pathological states such as diabetes. This is in marked contrast with glucagon levels observed in the GCGR global KO mice (>3,648 pM) (36) and in the liver-specific GCGR KO (~861 pM) (16). The reduction of hepatic *Gcgr* expression in αRheb^Tg^ mice was associated with downregulation of gluconeogenic and glycolytic enzymes, such as *Pepck*, *Gck,* and *Crtc2* (Figure 5A). Consistent with the differences in hepatic *Gcgr* expression among αRheb^Tg^, αTSC2^KO^, and GCGR-null mice, the downregulation of gluconeogenic and amino acid metabolism genes was not as extensive in αRheb^Tg^ mice as described for αTSC2^KO^ and GCGR-deficient mice, suggesting that glucagon resistance across a dose-response range on different downstream targets depends on the magnitude of the reduction in glucagon signaling. More importantly, normalization of glucagon levels in αRheb^Tg^ mice for 4 weeks was sufficient to restore liver *Gcgr*, *Pepck,* and *Gck* expression to normal levels (Figure 5A) without affecting the expression of genes regulating amino acid and urea cycle metabolism (Figure 5, B and C). The selective effect of normalization of glucagon levels on the expression of gluconeogenic genes without affecting other *Gcgr* downstream targets may suggest that part of the genetic alterations induced by chronic hyperglucagonemia are irreversible or that the recovery following glucagon reduction takes a longer period of time. Finally, the published evidence suggests that there is a dose response in the downregulation of gluconeogenesis, amino acids, and lipid metabolism by the magnitude of reduction in *Gcgr* expression and glucagon signaling. Extreme glucagon resistance obtained by global or liver-specific glucagon receptor deficiency shows marked alterations in gluconeogenesis and lipid and amino acid metabolism with hyperaminoacidemia. In contrast, the decrease in hepatic *Gcgr* expression in the αRheb^Tg^ mice is associated with a gradual decrease in glucagon target functions such as glucose levels, gluconeogenesis, and genes involved in gluconeogenesis and amino acid metabolism.

Glucagon action also plays a critical role in amino acid metabolism by regulating amino acid uptake in the liver, amino acid catabolism, and urea production. Inhibition or KO of *GCGR* decreases amino acid uptake, hyperaminoacidemia, and catabolism in the liver (18). Indeed, hepatic GCGR downregulation in αRheb^Tg^ mice was associated with decreases in *Got1*, *Pc*, *Sds,* and *Gpt1* mRNA in fasted αRheb^Tg^ mice, and those genes did not return to control levels after normalization of glucagon levels and *Gcgr* expression in αRheb^Tg^+Dox (Figure 5B). Postreceptor mechanisms regulating amino acid metabolism may require a longer time to recover after normalization of hyperglucagonemia and the increased *Gcgr* expression. Examination of urea production genes demonstrated a reduction of mRNA expression in only 1 urea production gene, *Oat* (Figure 5C). These results are in marked contrast to the reduced mRNA expression of the majority of urea production in models of GCGR deficiency and treatment with GCGR antagonist and suggest that the magnitude of the effects on urea production genes is proportional to the magnitude of reduction in GCGR expression and signaling. The results of the current and published studies (15, 17, 18, 24) suggest that the regulation of gluconeogenesis, amino acid metabolism, and urea production is sensitive to different levels of GCGR signaling activation. This hypothesis could be tested by assessing gluconeogenic, amino acid metabolism, and urea production genes in mice with heterozygous deletion of GCGR or mice with chronic infusion of glucagon at different concentrations.

This study supports the notion that glucagon and inhibition of glucagon receptor signaling can be used as a strategy to control hyperglycemia in diabetes. Glucagon/GLP-1 dual agonism is considered for the treatment of obesity. Our findings suggest that sustained activation of the glucagon receptor does not lead to hyperglycemia. The metabolic alterations induced by prolonged hyperglucagonemia are transient and reversible.

## Methods

### Animals and procedures.

Mice were housed in a pathogen-free environment and maintained on 12-hour-light/dark cycle at the University of Miami Facility. The Glucagon-Cre mice (obtained in-house) (37) expressing *Cre* recombinase driven by the glucagon promoter were crossed with Rheb^Tg^ mice to conditionally activate Rheb expression in α cells (αRheb^Tg^). The Rheb transgene was generated as described previously (38, 39). The Rheb transgene construct used to generate these mice was built on a backbone knockin single-cassette vector for the ROSA26 locus and contains the splice acceptor sequence, neomycin cassette, tTA gene (Tet-off), insulator sequence, CMV promoter responsive to tTA, rabbit β-globin intron, Rheb cDNA, internal ribosome entry site, and EGFP cDNA (Figure 1A). In this Tet-off model, expression of the tTA is turned on upon *Cre*-mediated recombination, and removal of the neo gene cassette induces Rheb expression in the absence of Dox. These mice had mixed background between C57BL/6 and 129X1. Rheb^Tg^ mice littermates were used as controls. This control group was selected after showing normal glucose tolerance in Glucagon-Cre mice, conserved glucose responses after insulin injection in αRheb^Tg^ mice receiving Dox chow (Supplemental Figure 1, A and D), and normal glucose tolerance and insulin tolerance in αRheb^TgHet^ mice (Glucagon-Cre/Rheb^Tg/+^) when compared with controls (Supplemental Figure 1, A–D). Littermate controls were used in all experiments to avoid potential effects from the genetic background. Islet morphometric analysis utilized age-matched cohorts with male and female mice. Dox was administered in chow diet (Dox 200 ppm, Research Diets, catalog D11071101).

### Metabolic studies.

To prevent Rheb overexpression during development, the breeders were fed Dox diet. The offspring were weaned on Dox diet until they were 1 month old. The removal of Dox diet allowed overexpression of Rheb upon *Cre*-mediated recombination under the glucagon promoter. Overnight fasting blood glucose and glucagon were monitored for 30 days after removal of Dox from the diet. Random fed (9 am) blood insulin was evaluated during the same period. The blood was obtained from the tail vein and blood glucose was measured with Accu-Chek blood glucose meter. IPGTT (2 g/kg) and ITT (0.75 U/kg) were performed by i.p. injections of respective agents in 6-hour and 4-hour-fasted male mice. Glucagon challenge was performed by i.p. injection of glucagon (100 μg/kg or 20 μg/kg; Sigma) in 6-hour-fasted male mice. We chose 100 μg/kg dose and 20 μg/kg to avoid the insulinotropic effect of glucagon induced by 1 mg/kg dose, and 100 μg/kg dose induces a greater glucose response in the control mice in previous studies (20, 40, 41).

### Islet studies.

Islet isolation was accomplished by collagenase digestion as described previously (19). Islets were cultured overnight in RPMI containing 5 mM glucose. Groups of 15 islets/mouse were placed in 8 μm cell culture inserts (Millicell), preincubated in HG KRBB (6 mM glucose) for 1–2 hours, and incubated subsequently for 1 hour in each condition: LG KRBB (2 mM glucose), HG KRBB (16.7 mM glucose), or HG KRBB+Diazoxide (Diaz) (200 μM) or HG KRBB+KCl (30 mM). Assessment of insulin content of the islets was performed by extraction in 0.5 mL acid-alcohol per 15 islets/insert after each assay. All assays represent results from 2 independent experiments. Secreted insulin levels and islet insulin content were measured with an ELISA (Alpco). All data are represented as secreted insulin in the culture medium normalized to islet insulin content for each insert of islets.

### Hormone and metabolite measurements.

Glucagon and insulin levels were measured with ELISAs (Mercodia [10 μL assay] and Alpco, respectively). Plasma urea levels were measured with the quantitative enzymatic Urea Assay Kit III (BioAssay Systems). Amino acids were measured with the L-Amino Acid Quantitation Kit (Sigma). All assays were performed according to manufacturer’s protocols. The plasma levels of active GLP-1 were measured with the STELLUX Chemiluminescent Assay (Alpco, catalog 80-GLP1A-CH01). Prior to measuring active GLP-1 levels, DPP-IV Inhibitor (Millipore) was added to plasma before storing the samples in –80°C.

### Flow cytometry and FACS.

Islets were isolated and incubated overnight in RPMI containing 6 mM glucose. The islets were dispersed into a single-cell suspension with trypsin-EDTA and fixed with BD Pharmingen Transcription Factor Phospho Buffer Set (BD Biosciences). The fixed cells were incubated with conjugated antibodies overnight at 4°C and gentle rotation. Dead cells were excluded by Ghost Dye Red 780 (Tonbo). Glucagon and pS6 (Ser 240) expression were analyzed by mean fluorescent intensity (MFI) per glucagon-positive cells using BD LSR II (BD Biosciences). The size of live glucagon-positive cells was analyzed by forward scatter area (FSC-A).

### Immunofluorescence and cell morphometry.

Pancreata were fixed in 3.7% formaldehyde, embedded in paraffin, and sectioned (5 μm). Fluorescent images were acquired using a microscope (Leica DM5500B) and a motorized stage using a camera (Leica DFC360FX) (Leica Microsystems). Cell mass was determined in 5 stained sections (5 μm) separated by 200 μm as described previously (19, 42, 43). The area of glucagon and the area of each section were quantified with NIH Image J Software (v1/49d). The ratio of the hormone-stained area to the total pancreatic section area for each mouse was averaged and multiplied by the pancreas weight. Antibody information is available in Supplemental Table 1.

### Quantitative real-time PCR.

For RNA expression, total RNA was extracted from liver samples using the RNeasy isolation kit (Qiagen). Gene expression was performed by quantitative real-time RT-PCR with Power SYBR Green PCR Mix (Applied Biosystems) using QuantStudio 3 Real-Time PCR systems (Applied Biosystems) with a standard protocol including a melting curve. Relative abundance for each transcript was calculated by a standard curve of cycle thresholds and normalized to 18S. Primers were purchased from IDT Technologies. Primer sequences are available in Supplemental Table 2.

### Western blotting.

After stimulation with insulin (1 U/kg) or glucagon (100 μg/kg) the liver was collected and homogenized in lysis buffer (125 mM Tris, pH 7, 2% SDS, 1 mM DTT) containing a phosphatase (Roche Diagnostics) and protease (Sigma) inhibitor cocktails. Homogenates were boiled for 5 minutes, loaded and electrophoresed on 4%–12% gradient SDS-PAGE gel, and transferred to polyvinylidene fluoride membranes. Phosphor-Akt (S473), Phosphor-CREB^(Ser133)^, total CREB and Cyclo B antibody were purchased from Cell Signaling. Antibodies used for immunoblotting are included in Supplemental Table 1, and membranes were developed using LI-COR Odyssey FC. Band densitometry was determined by measuring pixel intensity using NIH Image J software (v1/49d).

### Statistics.

The statistical analysis for comparisons between 2 groups was performed by unpaired (2-tailed) Student’s *t* test. One-way ANOVA with post hoc Dunnett’s multiple comparisons test was used for comparisons between 3 or more groups to common control. Two-way ANOVA with post hoc Šidák’s multiple comparisons test was used for comparisons among 3 or more groups without a common control. Statistical analysis was performed using GraphPad Prism 9.3. *P* values of less than 0.05 were considered significant.

### Study approval.

All protocols were approved by the University of Miami Animal Care and Use Committees and were in accordance with NIH guidelines.

### Data availability.

Reagents and genetically modified mice developed in the context of this manuscript will be shared with investigators from not-for-profit organizations who request them in accordance with institutional guidelines using a simple material transfer agreement.

## Author contributions

EBM conceived and designed experiments, analyzed results, and wrote the manuscript. EBM is the guarantor of this work. CL designed and performed the experiments, analyzed the results, and wrote the manuscript. NBK performed the experiments, analyzed the results, and wrote the manuscript. RAL performed the experiments, analyzed the results, and reviewed the manuscript. GL reviewed the manuscript. GKG generated mice. All authors contributed to the discussion and reviewed/edited the manuscript.

## Supplementary Material

Supplemental data

## Version 1 05/04/2023

In-Press Preview

## Version 2 06/08/2023

Electronic publication
