# Supplementary material for: Time-dependent effects of endogenous hyperglucagonemia on glucose homeostasis and hepatic glucagon action
Source: JCI Insight. 2023 Jun 8;8(11):e162255. doi: 10.1172/jci.insight.162255 (PMC10393226; doi:10.1172/jci.insight.162255)
Supplement: Supplemental data [file jciinsight-8-162255-s260.pdf]

Supplemental data

Supplemental figure legends:

Supplemental Figure 1. Normal glucose tolerance in Glucagon-Cre mice and conserved glucose responses after insulin injection in αRheb^{Tg} receiving Dox chow. A. Intraperitoneal glucose tolerance test in 4-month-old Glucagon-Cre (n=6) and littermate wild-type (WT) controls (n=13) mice. B. Intraperitoneal glucose tolerance test in 1-month-old Rheb and littermate Glucagon-Cre;Rheb$^{Tg\ Het}$ male mice. C. Insulin tolerance test in 3-month-old Rheb and littermate Glucagon-Cre;Rheb$^{Tg\ Het}$ male mice. **D.** Insulin tolerance test in Rheb^{Tg}+Dox (n=6) and αRheb^{Tg}+Dox (n=10) male mice at 4 weeks of age. Data are shown as means ± S.E.M.

Supplemental Figure 2: αRheb^{Tg} mice have decreased glucagon signaling with no changes in insulin secretion or insulin resistance. A. Glucagon challenge test (20 *u*g/kg) and **B.** AUC of glucagon challenge in 3-month-old Control mice (n= 6) and αRheb^{Tg} (n= 6). **C.** Exogenous glucagon (100 μg/kg) was directly injected into the portal vein of fasted control and αRheb^{Tg} anesthetized mice. Western blot of pCREB (Ser133) in liver lysates collected at baseline (fasted, 0 min) and 5- and 10-min post–glucagon injection. **D.** Insulin secretion in response to glucose (GSIS) and Diazoxide (200 μM) (Diaz) and in combination with KCl (30mM) (Diaz+KCl) in isolated islets from Control mice (n=10) and αRheb^{Tg} (n=10). **E.** Western blot and quantification showing hepatic insulin sensitivity measured by pAKT (Ser473) after intraperitoneal insulin administration (1 U/kg). Data are shown as means ± S.E.M. *$p<0.05$. (Student's 2-tailed t-test). For A data are shown as means ± S.E.M. *$p<0.05$ (two-way ANOVA with Sidak's post-test).

Supplemental Figure 3. Postnatal activation of Rheb 10 days after remove Dox.

A. % of alpha cell number determined by glucagon+ cell measured by flow cytometry in dispersed islets from control (n=6 mice) and αRheb^{Tg} (n=5 mice). **B**. Glucagon expression determined by the mean fluoresce intensity (MFI) in dispersed islets in glucagon+ cells from control (n=6 mice) and αRheb^{Tg} (n=6 mice). **C**. Assessment of pS6^{Ser240} by mean fluorescence intensity (MFI) measured by flow cytometry in dispersed alpha cells (n=6 mice) and αRheb^{Tg} (n=5 mice). **D**. α-cell size analyzed by flow cytometry using dispersed islets and quantified by forward scatter area (FSC-A) of control (n=6 mice) and αRheb^{Tg} (n=5 mice). **E**. RNA expression of hepatic key enzymes involved in gluconeogenesis in 6 hours fasted liver from control (n=6) and αRheb^{Tg} (n= 6) 10 days after removing Dox diet. Data are shown as means ± S.E.M. *$p<0.05$. (Student's 2-tailed *t* test).

Supplemental Figure 4. No changes in plasma urea and amino acid levels in control and αRheb^{Tg} mice. A. Body weight, **B.** Fasting (6 hours) urea levels, and **C.** Fasting (6 hours) amino acid levels from controls (n=6), αRheb^{Tg} (n=6) and αRheb^{Tg}+Dox (n=4) mice at 4 months of age. Data for A-D are shown as means ± S.E.M.

Supplemental Table 1. Antibodies

Supplemental Table 2. Primer Sequences

Supplemental Figure 1

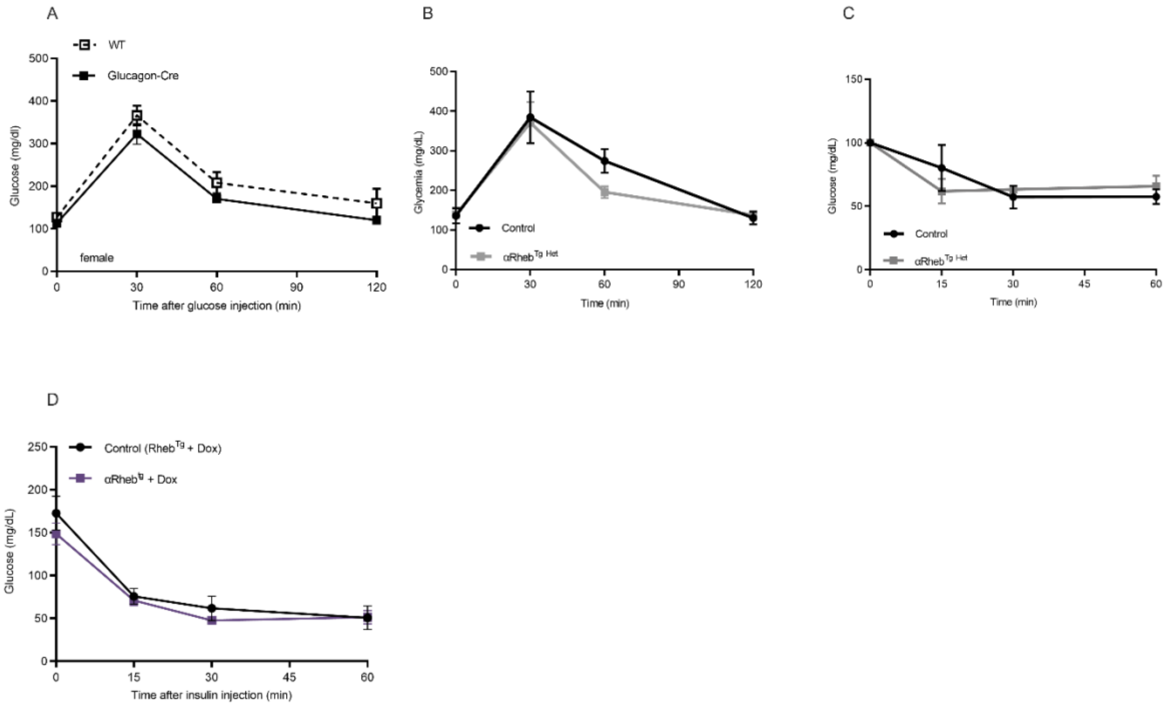

Supplemental Figure 1. Normal glucose tolerance in Glucagon-Cre mice and conserved glucose responses after insulin injection in αRheb^{Tg} receiving Dox chow. A. Intraperitoneal glucose tolerance test in 4-month-old Glucagon-Cre (n=6) and littermate wild-type (WT) controls (n=13) mice. B. Intraperitoneal glucose tolerance test in 1-month-old Rheb and littermate Glucagon-Cre;Rheb$^{Tg\ Het}$ male mice. C. Insulin tolerance test in 3-month-old Rheb and littermate Glucagon-Cre;Rheb$^{Tg\ Het}$ male mice. **D.** Insulin tolerance test in Rheb^{Tg}+Dox (n=6) and αRheb^{Tg}+Dox (n=10) male mice at 4 weeks of age. Data are shown as means ± S.E.M.

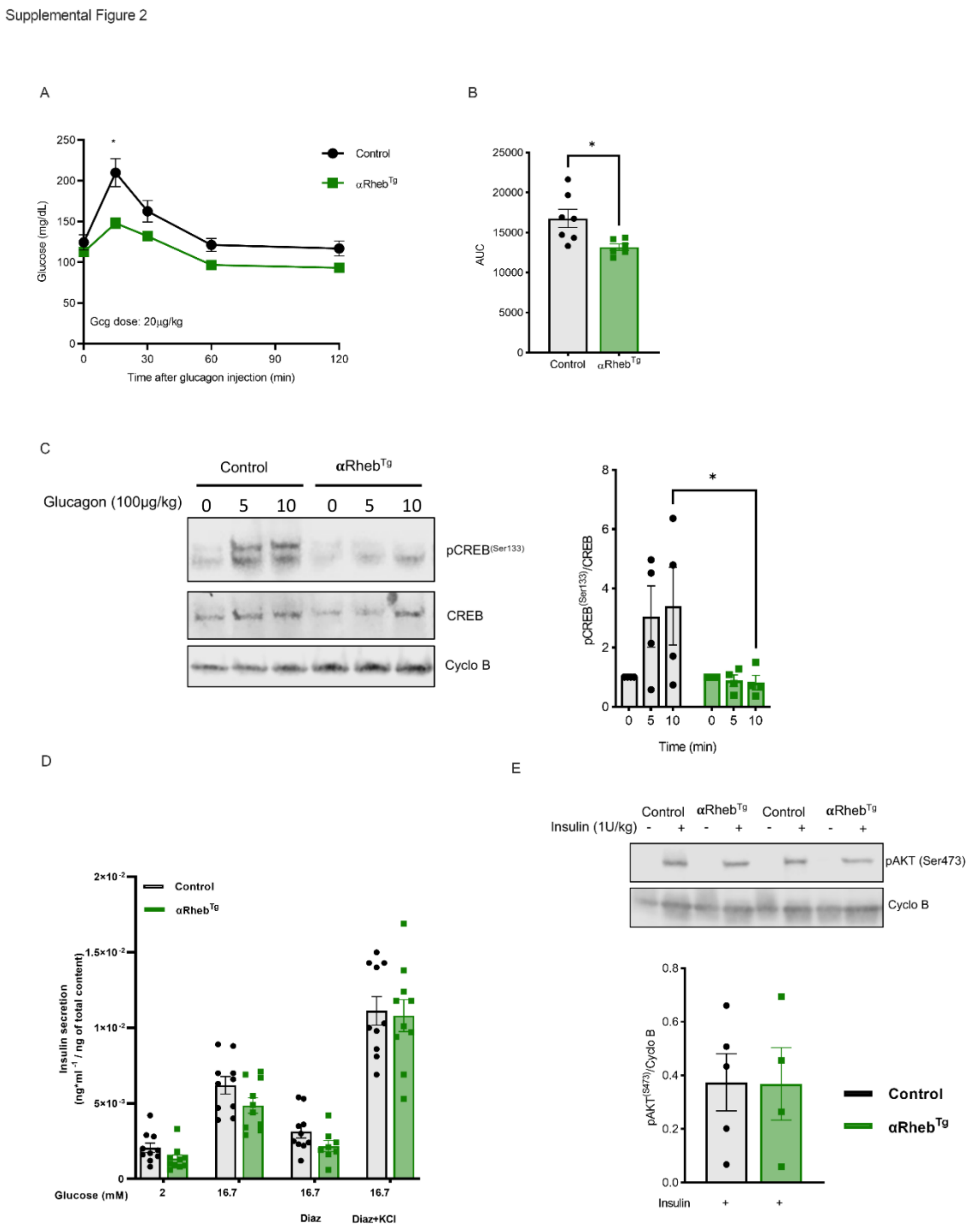

Supplemental Figure 2: αRheb^Tg mice have decreased glucagon signaling with no changes in insulin secretion or insulin resistance. A. Glucagon challenge test (20 *u*g/kg) and **B.** AUC of glucagon challenge in 3-month-old Control mice (n= 6) and αRheb^Tg (n= 6). **C.** Exogenous

glucagon (100 μg/kg) was directly injected into the portal vein of fasted control and α$Rheb^{Tg}$ anesthetized mice. Western blot of pCREB (Ser133) in liver lysates collected at baseline (fasted, 0 min) and 5- and 10-min post–glucagon injection. **D.** Insulin secretion in response to glucose (GSIS) and Diazoxide (200 μM) (Diaz) and in combination with KCl (30mM) (Diaz+KCl) in isolated islets from Control mice (n=10) and α$Rheb^{Tg}$ (n=10). **E.** Western blot and quantification showing hepatic insulin sensitivity measured by pAKT (Ser473) after intraperitoneal insulin administration (1 U/kg). Data are shown as means ± S.E.M. *p<0.05. (Student's 2-tailed *t* test). For A data are shown as means ± S.E.M. *p<0.05 (two-way ANOVA with Sidak's post test).

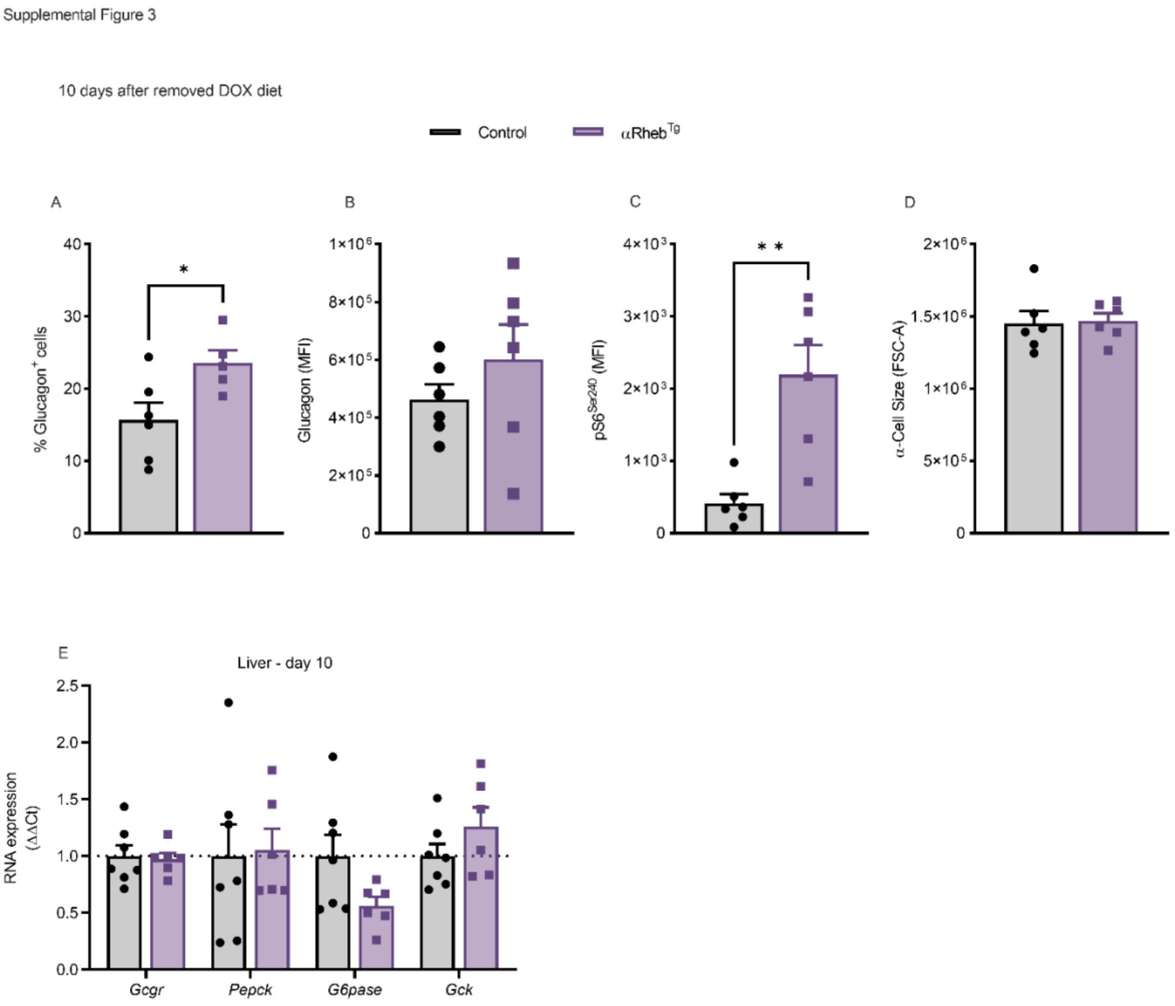

Supplemental Figure 3. Postnatal activation of Rheb 10 days after remove Dox.

A. % of alpha cell number determined by glucagon+ cell measured by flow cytometry in dispersed islets from control (n=6 mice) and αRheb^{Tg} (n=5 mice). **B**. Glucagon expression determined by the mean fluoresce intensity (MFI) in dispersed islets in glucagon+ cells from control (n=6 mice) and αRheb^{Tg} (n=6 mice). **C**. Assessment of pS6^{Ser240} by mean fluorescence intensity (MFI) measured by flow cytometry in dispersed alpha cells (n=6 mice) and αRheb^{Tg} (n=5 mice). **D**. α-cell size analyzed by flow cytometry using dispersed islets and quantified by forward scatter area (FSC-A) of control (n=6 mice) and αRheb^{Tg} (n=5 mice). **E**. RNA expression of hepatic key

enzymes involved in gluconeogenesis in 6 hours fasted liver from control (n=6) and αRheb^{Tg} (n=6) 10 days after removing Dox diet. Data are shown as means ± S.E.M. *$p<0.05$. (Student's 2-tailed *t* test).

Supplemental Figure 4

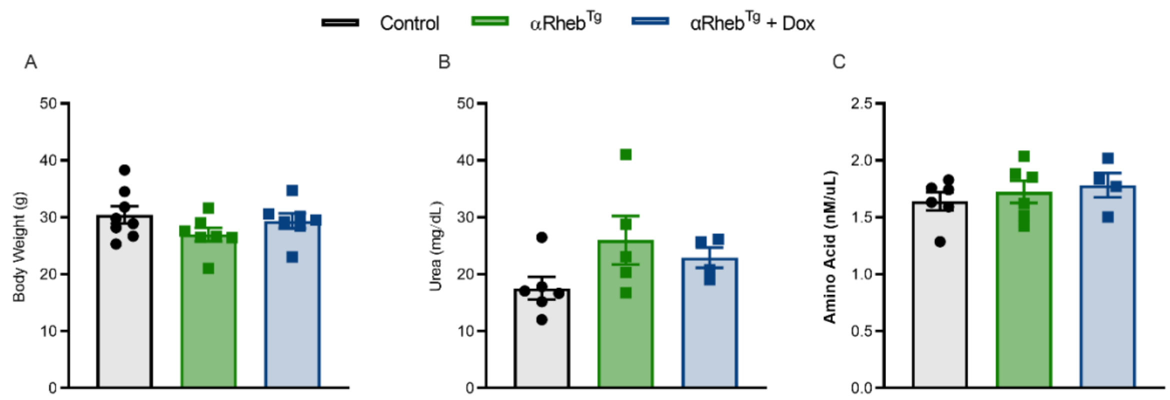

Supplemental Figure 4. No changes in plasma urea and amino acid levels in control and αRheb^{Tg} mice. A. Body weight, **B.** Fasting (6 hours) urea levels and **C.** Fasting (6 hours) amino acid levels from controls (n=6), αRheb^{Tg} (n=6) and αRheb^{Tg}+Dox (n=4) mice at 4 month of age. Data for A-D are shown as means ± S.E.M.

Supplemental Table 1. Antibodies

**Antibody**	**Specie**	**Catalog number**	**Source**	**Application**
Creb	Mouse	9104S	Cell signaling	WB
Cyclo B	Rabbit	PA1027A	Thermo Fisher	WB
Glucagon	Mouse	Ab10988	Abcam	IFC
Glucagon	Rabbit	Ab932	EMD Millipore	IFC
Glucagon-BV421	Human/Mouse	565891	BD Biosciences	FC
Insulin	Guinea Pig	ir002	Dako	IFC
Insulin-APC	Human/Mouse/Bovine	IC1417A	R&D Systems	FC
pAkt (Ser473)	Rabbit	4060S	Cell signaling	WB
pCreb (Ser133)	Rabbit	9198S	Cell signaling	WB
pS6 (S240) – PE600	Mouse	560430	BD Biosciences	FC
Ghost Dye Red 780		13-0856-T500	Tonbo	FC

IFC = Immunofluorescent Chemistry

FC=Flow Cytometry

Supplemental Table 2. Primer Sequences

**Gene**	**Forward**	**Reverse**
Cps1	CATGGAACATCCAGCCGAATTGG	CATGGAACATCCAGCCGAATTGG
Gcgr	ACGGTACAGCCAGAAGAT	CTACCAGCAACCAGCAATAG
Pepck	ATCATCTTTGGTGGCCGTAG	ATCTTGCCCTTGTGTTCT
G6pase	CCGGTGTTTGAACGTCATCT	CAATGCCTGACAAGACTCCA
Gck	CTGTTAGCAGGATGGCAGCTT	TTTCCTGGAGAGATGCTGTGG
Fas	AGAAGCCATGTGGGGAAGATT	AGCAGGGACAGGACAAGACAA
Sds	CACTGGCCTCGCTGGTTGTCATT	GTGGCCAGGGCAGCAGCAGAT
Gpt1	CTTTGAAAGCAGTGCAGCGT	AAATCAGGCCTACACCCAGC
Pc	ATCCAGCGGCGGCACCAGA	GCGGGAATTGACCTCGATGAAGTA
Got1	TCTGACCGTGGTCGGAAAAG	TTTGGTGGCGTGAACTACGA
Crtc2	GGTGGTTCTCTGCCCAATGT	AGTCAGAGCTTGTCCTTCGC
Arg1	TATGACGTGAGAGACCACGG	CTTCCAACTGCCAGACTGTG
Oat	TGGCGGTTTATACCCTGTGT	AAACCTCAAGAGCCGCAATG
Ass1	GAGTGGGTCGCATTGACATC	GGGTCTCGTAGATACCTCGG
Nnmt	ATATTCTGCCTGGGTGCTGT	AGGCTCCTGGTTCCTTCTTC
Fgf21	AGATCAGGGAGGATGGAACA	TCAAAGTGAGGCGATCCATA
18S	GCAATTATTCCCCATGAACG	GGGACTTAATCAACGCAAGC
